# Characterization of a Novel Single-Chain Bispecific Antibody for Retargeting of T Cells to Tumor Cells via the TCR Co-Receptor CD8

**DOI:** 10.1371/journal.pone.0095517

**Published:** 2014-04-21

**Authors:** Irene Michalk, Anja Feldmann, Stefanie Koristka, Claudia Arndt, Marc Cartellieri, Armin Ehninger, Gerhard Ehninger, Michael P. Bachmann

**Affiliations:** 1 Institute of Immunology, Medical Faculty ‘Carl Gustav Carus’, TU Dresden, Dresden, Germany; 2 Helmholtz Zentrum Dresden-Rossendorf, Institute of Radiopharmaceutical Cancer Research, Department of Radioimmunology, Dresden, Germany; 3 Medical Clinic and Polyclinic I, University Hospital ‘Carl Gustav Carus’, TU Dresden, Dresden, Germany; 4 Center for Regenerative Therapies Dresden, TU Dresden, Dresden, Germany; New York University, United States of America

## Abstract

There is currently growing interest in retargeting of effector T cells to tumor cells via bispecific antibodies (bsAbs). Usually, bsAbs are directed on the one hand to the CD3 complex of T cells and on the other hand to a molecule expressed on the surface of the target cell. A bsAb-mediated cross-linkage via CD3 leads to an activation of CD8+ T cells and consequently to killing of the target cells. In parallel, CD4+ T cells including TH_1_, TH_2_, TH_17_ cells and even regulatory T cells (Tregs) will be activated as well. Cytokines produced by CD4+ T cells can contribute to severe side effects e. g. life-threatening cytokine storms and, thinking of the immunosupressive function of Tregs, can even be counterproductive. Therefore, we asked whether or not it is feasible to limit retargeting to CD8+ T cells e. g. via targeting of the co-receptor CD8 instead of CD3. In order to test for proof of concept, a novel bsAb with specificity for CD8 and a tumor-associated surface antigen was constructed. Interestingly, we found that pre-activated (but not freshly isolated) CD8+ T cells can be retargeted via CD8-engaging bsAbs leading to an efficient lysis of target cells.

## Introduction

Since the development of the hybridoma technology a series of problems became evident which limit the clinical use of monoclonal antibodies (mAbs). One major disadvantage of murine mAbs is their inefficient triggering of human effector functions including the complement system and antibody-mediated cellular cytotoxicity. Therefore, over the past decades a series of ideas were put forward to enhance cytotoxic effects of murine mAbs in order to improve their benefit especially in tumor therapy. For example, toxic compounds including radioactive isotopes were linked to mAbs for delivery to tumor cells [e. g. 1, 2]. However, even until today the number of clinically used mAbs is still small. Another approach to enhance killing efficiency of murine mAbs is based on the idea to cross-link effector cells with target cells using bispecific Abs (bsAbs). Originally, bsAbs were obtained by chemical cross-linkage or by the quadroma technology [e. g. 3]. Although the only approved bi/trispecific mAb catumaxomab so far is produced by quadroma technology, this technology like many others appears to have a series of drawbacks. On the one hand, quadromas are formed by fusion of two hybridoma cell lines. As a consequence, both heavy and light chains are combined randomly. Thus, only a limited portion of quadroma-produced bsAbs has the desired specificity. Moreover, as the quadroma cell is derived from a mouse and a rat hybridoma cell the resulting bsAb is immunogenic in humans and its application is limited due to the formation of human anti-mouse Abs (HAMAs). Recombinant Ab technologies finally helped to achieve the breakthrough of bsAbs. However, it still took more than a decade and a plethora of constructs had to be created from a long list of investigators until highly efficient and sufficiently stable bsAbs became available that are currently on the way into the clinics [e. g. 4, 5]. Especially single-chain bsAbs represent promising therapeutic molecules [4]–[6]. Such bsAbs are usually generated by fusion of the minimal binding domains (Fv, fragment variable) of two mAbs. By simultaneous binding to the activating CD3 complex and a tumor-associated surface antigen (TAA), such bsAbs (also known as BiTEs for bispecific T cell engagers) are able to trigger a T cell-mediated tumor cell lysis in a T cell receptor (TCR)- and MHC-independent manner [6]–[11]. Their highly efficient antitumor activity has already been shown both *in vitro* and in animal studies [4], [5]. First clinical trials with blinatumomab, the first BiTE successfully applied for treatment of B cell leukemia and lymphoma patients, support their functionality even in men [11]. As the CD3 complex assembles with all TCRs BiTEs are able to cross-link target cells not only with CD8+ cytotoxic T cells but also with CD4+ T cells including TH_1_, TH_2_, TH_17_ and even regulatory T cells (Tregs). It is commonly known that activation of CD4+ T cells results in the release of huge amounts of cytokines and thereby can contribute to life-threatening cytokine storms. Moreover, it has already been shown by our group that the suppressive mechanisms of Tregs can be triggered after bsAb-mediated cross-linkage to tumor cells [e. g. 12]. In order to circumvent the activation of CD4+ T cells we, therefore, tried to develop tools for selective retargeting of CD8+ T cells. For proof of concept, we constructed a novel bsAb with specificity for the co-receptor CD8 of the TCR complex and for prostate stem cell antigen (PSCA) as one potential TAA. Here we show that pre-activated CD8+ T cells can be efficiently redirected via CD8-engaging bsAbs for killing of tumor cells.

## Results

### Construction and Purification of a Novel bsAb for Retargeting of T Cells via the Co-receptor CD8

**Figure 1:**
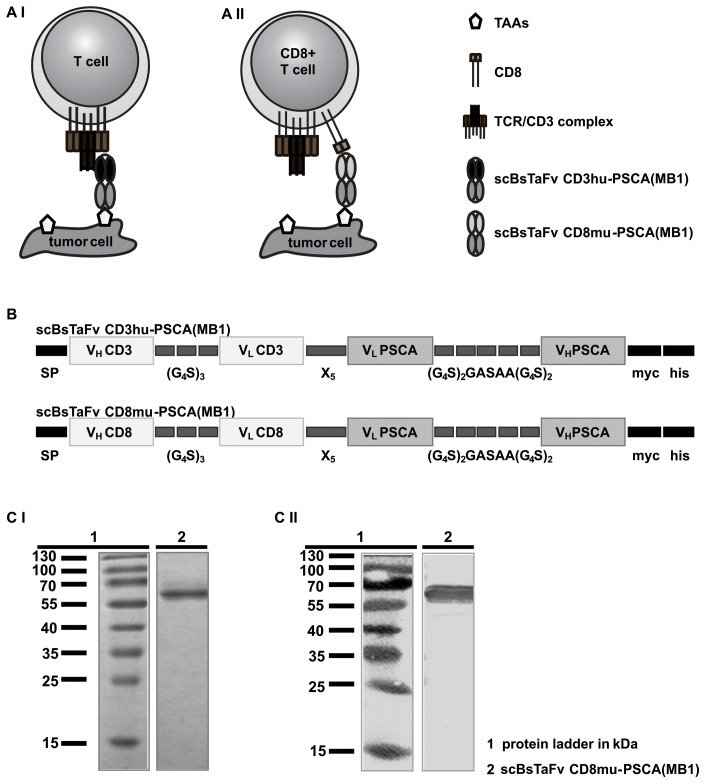
Comparison of conventional CD3-engaging bsAbs with CD8-engaging bsAbs with regard to (A) principal idea, (B) schematic structure, (C) production and purification. *A*, cross-linkage of T cells and tumor cells can be mediated by conventional bsAbs via simultaneous binding to the CD3 part of the TCR/CD3 complex and a tumor-associated surface antigen (TAA) (*AI*). A subtype-specific cross-linkage of CD8+ T cells can theoretically be achieved by a CD8-engaging bsAb (*AII*). *B*, schematic structure of recombinant single-chain bsAbs. For construction of the novel CD8-engaging single-chain bsAb, the anti-CD3 domain of scBsTaFv CD3-PSCA(MB1) was replaced by the scFv derived from the novel anti-CD8 mAb clone MB10. Recombinant Ab constructs were further equipped N-terminally with an Igκ leader as signal peptide (SP) for Ab secretion and C-terminally with a myc- and a his-tag for protein purification and detection. *C*, recombinant Abs were purified via Ni-NTA affinity chromatography from cell culture supernatant. Purified bsAbs were analyzed by SDS-PAGE and stained with Coomassie Brilliant Blue G250 to estimate protein purity and concentration (*CI*). Purified bsAbs were further analyzed by immunoblotting (*CII*). After transfer onto nitrocellulose membranes recombinant Abs were detected via their C-terminal his-tag.

As schematically summarized in **Fig. 1AI** conventional single-chain bsAbs for retargeting of T cells to tumor cells are directed on the one hand to the CD3 complex of the TCR complex and on the other hand to a surface target antigen. In previous studies we among others have established such highly active single-chain bsAbs for redirection of T cells to PSCA+ tumor cells [13]–[15]. In order to recruit only the CD8+ T cell subpopulation for tumor cell killing, we here generated for proof of concept a novel CD8-directed single-chain bsAb by combination of the same functional anti-PSCA domain (clone MB1) with an Ab domain directed to CD8 (**Fig. 1AII**). In a first step, mAbs recognizing the human CD8 molecule were established by conventional hybridoma technology. After subcloning of the hybridoma cells the variable heavy (V_H_) and light chain (V_L_) sequences of a selected clone were determined and a single-chain fragment variable (scFv) in the orientation V_H_–V_L_ was constructed. Next, we replaced the CD3 domain in the previously described single-chain bispecific tandem fragment variable (scBsTaFv) CD3-PSCA(MB1) with the newly generated anti-CD8 scFv. The structure of the resulting novel single-chain bsAb compared to the original scBsTaFv CD3-PSCA(MB1) is schematically summarized in **Fig. 1B**. It is noteworthy to mention that in all bispecific constructs we used the same kind and sizes of glycine-serine (GS)-linker elements. Moreover, all heavy and light chain sequences were organized in the same orientation. For secretion into cell culture supernatants all constructs were equipped with an N-terminal signal peptide (**Fig. 1B**, SP). For purification and detection, a myc- and 6×histidine (his)-Tag was fused to the C-terminus (**Fig. 1B**, myc, his).

The novel recombinant scBsTaFv CD8-PSCA(MB1) was purified from cell culture supernatants by Ni-NTA affinity chromatography and isolated proteins were analyzed by SDS-PAGE (**Fig. 1C**, CI) and immunoblotting (**Fig. 1C**, CII). As shown in **Fig. 1C**, the purified recombinant Ab was sufficiently enriched after affinity chromatography, had the estimated molecular weight of approximately 60 kDa and could be detected with an anti-his Ab.

### Characterization of the Novel Single-chain bsAb for Retargeting of T Cells via the Co-receptor CD8

**Figure 2:**
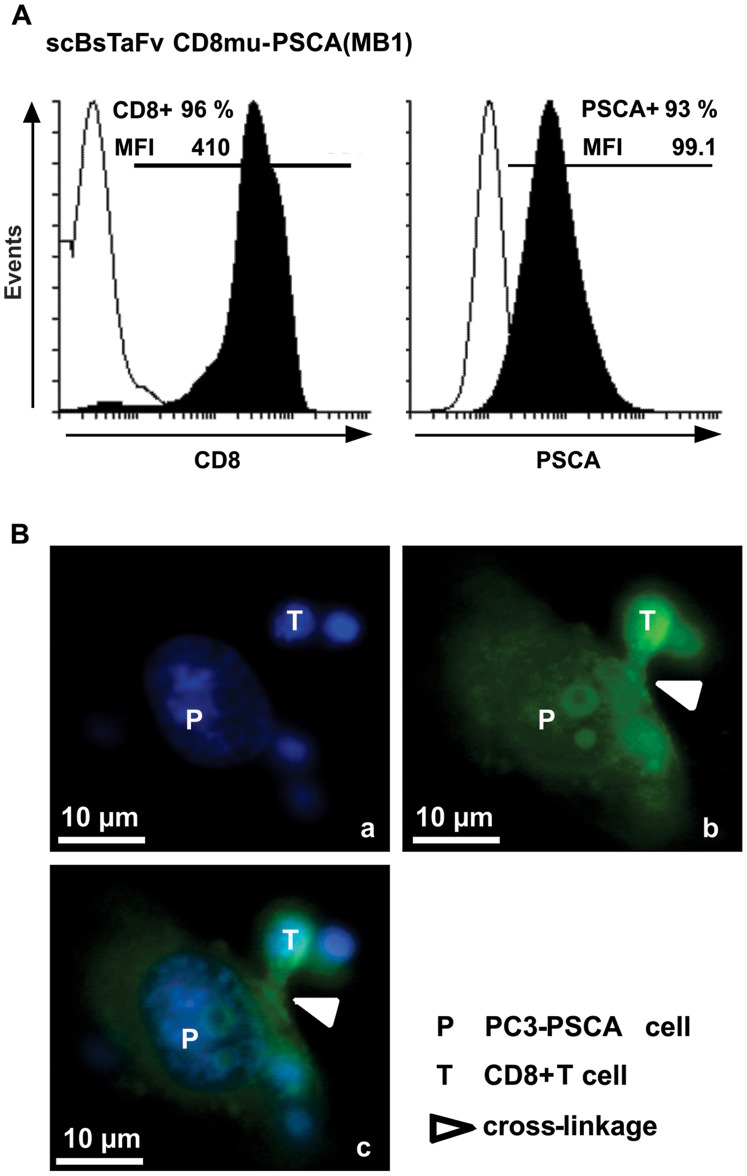
Binding properties of the novel scBsTaFv CD8-PSCA(MB1). *A*, in order to investigate binding properties of the novel single-chain bsAb, PC3-PSCA cells and isolated CD8+ T cells were stained with 10 ng/µl of recombinant Ab. Specific binding of the scBsTaFv CD8-PSCA(MB1) was detected with anti-myc/FITC. Histograms show percentage and mean fluorescence intensity (MFI) of stained antigen-positive cells (*black*) in comparison to the negative control incubated only with the secondary anti-myc/FITC mAb (white). *B*, to demonstrate simultaneous binding of the novel scBsTaFv CD8-PSCA(MB1) to PC3-PSCA cells and CD8+ T cells and, hence, to visualize a cross-linkage between the two cell types, microscopic images were taken. Therefore, PC3-PSCA cells and isolated CD8+ T cells were co-cultivated in the presence of scBsTaFv CD8-PSCA(MB1) for 22 h and fixed with 90% methanol. The scBsTaFv CD8-PSCA(MB1) was detected with anti-myc/FITC mAb (*green*) and cell nuclei were stained with DAPI (*blue*) containing sample cover medium. Microscopic image (*a*) shows DAPI-stained nuclei of one PC3-PSCA cell (*P*) surrounded by four T cells (*T*). A homogenous cell surface staining of the PC3-PSCA cell and T cells is shown in picture (*b*) after detection of the scBsTaFv CD8-PSCA(MB1) with anti-myc/FITC. Furthermore, a cross-linkage between the PC3-PSCA cell and a T cell is visible (*white triangle*). Image (*c*) is an overlay of (*a*) and (*b*).

Using flow cytometry analysis we tested whether or not each of the two arms of the novel single-chain bsAb is capable of binding to its respective target site. As shown in **Fig. 2A** the scBsTaFv CD8-PSCA(MB1) was not only able to bind to PSCA+ target cells but also to CD8+ T cells. As well as the maternal anti-CD8 mAb the bispecific construct failed to bind to CD4+ T cells (data not shown). Furthermore, T cells could be cross-linked to target cells with the help of the scBsTaFv CD8-PSCA(MB1) as verified by epifluorescence microscopy (**Fig. 2B**). Observed T cell/target cell synapses were similar to those mediated by conventional CD3-directed bsAbs [11] but did not occur in the absence of any bsAb (data not shown).

### BsAb-mediated Cross-linkage of Target Cells with Freshly Isolated Non-activated T cells via CD8 is not Sufficient for T cell Activation

**Figure 3:**
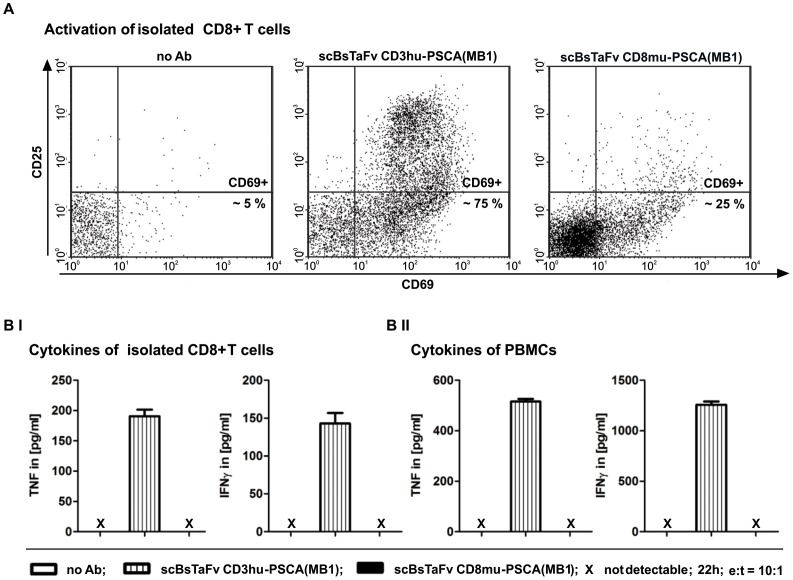
Activation and cytokine release of freshly isolated CD8+ T cells or PBMCs after cross-linkage to PC3-PSCA cells via the novel scBsTaFv CD8-PSCA(MB1). *A*, freshly isolated CD8+ T cells were cultured with PC3-PSCA cells in the absence or presence of 30 pmol/ml bsAb at an e:t ratio of 10∶1 for 24 h. Up-regulation of activation markers CD69 and CD25 on T cells was measured by flow cytometry and is shown for one representative donor. Numbers in dot plots represent percentage of CD8+/CD69+ cells. *B*, cell culture supernatants from co-cultures of T cell activation experiments (*A*) were collected to determine concentrations of IFN-γ (*left*) or TNF (*right*) by ELISA. The activation experiments were additionally performed under the same conditions as already described but using freshly isolated PBMCs instead of isolated CD8+ T cells. For technical reasons isolated T cells and pre-activated PBMC samples were from independent donors. Data of one representative CD8+ T cell donor (*BI*) and one representative PBMC donor (*BII*) are shown.

Our previous studies have shown that cross-linkage of freshly isolated T cells with PSCA+ tumor cells via the CD3 complex using the scBsTaFv CD3-PSCA(MB1) results in efficient activation of both CD8+ and CD4+ T cells. In agreement with this, co-cultivation of non-activated CD8+ T cells with PC3-PSCA cells in the presence of the scBsTaFv CD3-PSCA(MB1) leads to a substantial upregulation of the activation markers CD25 and CD69 (**Fig. 3A** **, middle**). In contrast, but not unexpected, no considerable activation of T cells was observed upon engagement via the co-receptor CD8 (**Fig. 3A** **, right**). Although CD69 was upregulated to a small extend in the presence of the scBsTaFv CD8mu-PSCA(MB1), the CD25 expression level of redirected CD8+ T cells did not increase. In line with this lack of activation, no significant amounts of cytokines could be detected in co-culture supernatants after cross-linkage of non-activated CD8+ T cells or non-activated peripheral blood mononuclear cells (PBMCs) with target cells via the co-receptor CD8 (**Fig. 3BI, BII**). In contrast, T cells engaged via the CD3 complex produced significant amounts of pro-inflammatory cytokines including TNF and IFN-γ as a consequence of bsAb-mediated activation (**Fig. 3BI, BII**).

### BsAb-mediated Cross-linkage of Target Cells with Pre-activated T cells via the Co-receptor CD8 Induces Efficient Tumor Cell Killing

**Figure 4:**
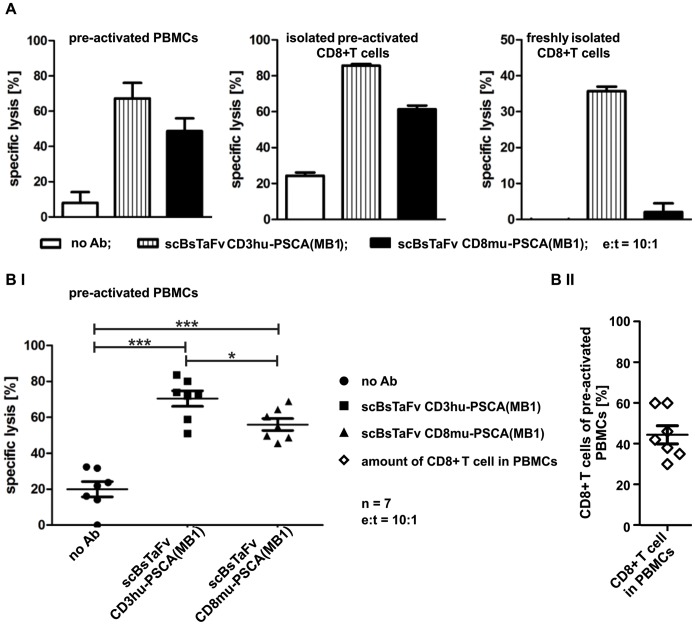
Tumor cell elimination mediated by the novel scBsTaFv CD8-PSCA(MB1). To analyze killing properties of the novel CD8-engaging single-chain bsAb standard chromium release assays were performed. *A*, to compare the anti-tumor effect of the novel scBsTaFv CD8-PSCA(MB1) with the conventional bsAb CD3-PSCA(MB1), [^51^Cr]-labeled PC3-PSCA cells and either pre-activated PBMCs (*left panel*), isolated pre-activated CD8+ T cells (*middle panel*) or freshly isolated CD8+ T cells (*right panel*) were cultivated in the presence or absence of 30 pmol/ml of recombinant bsAbs for 22 h. Mean of specific lysis ± SD of one representative donor is shown. *BI*, summary of (*A, left panel*) seven different donors is shown. Statistical significance was determined with one-way ANOVA and Bonferroni Multiple Comparison test (***p<0.001 with respect to control: no Ab; *p<0.05 significant difference between the conventional anti-CD3 bsAb and the novel anti-CD8 bsAb). *BII,* In order to estimate the ratio of CD8+ to CD4+ T cells, the pre-activated PBMC preparations of all seven donors were stained for CD8+ and CD4+ T cells and analyzed by FACS.

As freshly isolated CD8+ T cells could not be activated via the scBsTaFv CD8-PSCA(MB1), we finally tested whether or not pre-activated CD8+ T cells can be engaged via the co-receptor CD8 for an efficient bsAb-mediated tumor cell lysis. For this purpose, PSCA+ target cells were incubated with either prestimulated PBMCs (mainly composed of CD8+ T cells) or bead-activated isolated CD8+ T cells in the presence or absence of the scBsTaFv CD8-PSCA(MB1) or the scBsTaFv CD3-PSCA(MB1). Interestingly, retargeting of pre-activated PBMCs as well as isolated pre-activated CD8+ T cells resulted in efficient lysis of PSCA+ target cells in the presence of the scBsTaFv CD8-PSCA(MB1) (**Fig. 4A** **,BI**). However, lysis of target cells required relatively high e:t ratios of 10 to 1. The ratio of CD8+ to CD4+ T cells in the seven pre-activated PBMC samples varried between 30% and 60% with a mean value of 45% (**Fig. 4BII**).

As shown in Fig. 4B(I), killing effects mediated by the scBsTaFv CD8-PSCA(MB1) were significantly lower in comparison to the conventional CD3-engaging scBsTaFv CD3-PSCA(MB1) (Fig. 4BI). These differences might be attributed to the effector cell population (pre-stimulated PBMCs) investigated which still contains CD4+ T cells that can be engaged for tumor cell lysis via the scBsTaFv CD3-PSCA(MB1) but not via the scBsTaFv CD8-PSCA(MB1)).

## Discussion

In order to overcome limited effector functions of murine mAbs a series of recombinant bsAb formats have been developed over the past two decades [e. g. 4, 5]. All of them have their own advantages and disadvantages. Encouraging clinical responses were observed in recently published clinical trials, in which T cells were redirected using the BiTE blinatumomab for treatment of some malignancies of B cell origin [e. g. 7–9, 11]. Commonly, BiTEs engage T cells via the activating CD3 complex of the TCR complex. The cross-linkage to antigen-positive target cells leads to a capping effect at the T cell/target cell contact site. The formation of this immune synapse-like interaction [e. g. 5, 8, 10] is sufficient for activation of the T cells and efficient killing of target cells. Surprisingly, no additional co-stimulatory signal appears to be required [16]. Controversial data had been reported with respect to the contribution of CD4+ T cells to elimination of target cells after cross-linkage via CD3-engaging bsAbs [8], [13]–[18]. Recently, we could explain these discrepancies as a result of the respective experimental setting. It emerged from these studies that CD8+ T cells are able to start killing immediately after cross-linkage to a target cell via a bsAb [13], [15]. In contrast to CD8+ T cells, CD4+ T cells show a lag phase of about four to five hours before they acquire fully functional killing capabilities. Consequently, a contribution to killing of target cells by CD4+ T cells can hardly be detected if the killing assay is limited to this time frame [e. g. 13, 15]. The reason for the observed delay may be that professional CD8+ killer T cells are already equipped with cytotoxic molecules such as perforin and granzymes while these molecules are normally not required for the physiological function of CD4+ T cells and therefore need to be synthesized in order to convert CD4+ T helper cells into CD4+ killer T cells. This interpretation is in good agreement with previously published data by Grossman et al. [20] who report that about 50% of CD8+ peripheral T cells express granzyme A and B while only few CD4+ naive T cells were tested positive for these enzymes. Moreover, granzyme B expression was induced in CD4+ T cells after activation via anti-CD3/anti-CD28 Abs.

On the one hand, a contribution of CD4+ T cells to killing of target cells may have obvious advantages. On the other hand, a CD3-engaging bsAb will mediate a polyclonal activation of all subclasses of CD4+ T cells including Tregs. Aside from the suppressive effect of Tregs unwanted high amounts of cytokines will be released by activated CD4+ T effector cells which increase the risk of cytokine storms. Therefore, we thought of alternative ways to selectively redirect only CD8+ cytotoxic T cells. According to our current understanding, CD8 co-receptor molecules are normally outside of but assemble with the CD3/TCR complex after interaction of the TCR with a peptide:MHC complex. Thus, a simple cross-link between a T cell and a target cell via the CD8 co-receptor should not be sufficient to result in a CD3-based signaling cascade within a T cell which is necessary for conversion of T cells into activated effector T cells. However, we speculated that pre-activation of T cells by interleukin 2 (IL-2) in combination with anti-CD3/anti-CD28-coated beads and subsequent co-receptor-mediated cross-linkage to tumor cells could perhaps already be operative. Indeed, we found that bsAb-mediated cross-linkage of non-activated T cells via the co-receptor CD8 is not sufficient to trigger T effector cell functions but the combination of pre-activation and co-ligation leads to an efficient and selective tumor cell killing. The killing mediated by the anti-CD8 based bsAb appears to be less efficient compared to the anti-CD3 based bsAb. The reasons for these differences remain unclear. There are a series of possible explanations: For an efficient killing the affinities of a bsAb towards both the effector- and the target cell must be optimally balanced for repeated binding and release which may not be true for the currently available anti-CD8 based bsAb. Different affinities towards the effector cell side may influence the interaction time interval between binding and release of the effector cell to the target cell which will directly influence the efficacy of killing. Moreover, the two bsAbs are targeting different signalling pathways. Furthermore, the CD8 co-receptor is normally not part of the T cell receptor complex. So it has to be translocated into this complex in order to (indirectly) lead to the killing signal of the T cell receptor complex. As CD3 is not only part of the T cell receptor complex but directly involved in the creation this activation signal, a translocation of CD8 into this complex may be incomplete and therefore less efficient compared to the direct signalling via the CD3 complex. Nonetheless, the here described subclass specific targeting opens the chance to specifically redirect CD8+ T cells in cancer patients either by adoptive transfer of *ex vivo* activated CD8+ T cells combined with CD8-engaging bsAb treatment or by application of a CD8-directed bsAb in combination with IL-2 treatment.

In summary, we created a novel single-chain bsAb for retargeting of T cells via the co-receptor CD8. Thereby, we were able to show that pre-activated CD8+ T cells can indeed be redirected to target cells via their CD8 co-receptor resulting in an efficient killing of the target cell.

## Materials and Methods

### Ethics Statement

Human PBMCs were isolated either from buffy coats supplied by the German Red Cross (Dresden, Germany) or from fresh blood of healthy donors with their written consent. The study including the consent form was approved by the local ethics committee of the Medical Faculty of the University Hospital ‘Carl Gustav Carus’ TU-Dresden (EK27022006).

### Isolation and Cell Culture of Human PBMCs and T Cell Fractions

Human PBMCs were isolated from either fresh blood of healthy donors or buffy coats supplied by the German Red Cross (Dresden, Germany) by gradient centrifugation over Biocoll (Biochrom, Berlin, Germany) and cultivated as previously described [e. g. 14, 15]. CD8+ T cells were purified from PBMCs by negative selection using the CD8+ T cell Isolation Kit (Miltenyi Biotec, Bergisch Gladbach, Germany). PBMC activation was performed as described in [e. g. 14]. Isolated CD8^+^ T cells were expanded using DynaBeads (Invitrogen, Karlsruhe, Germany) at a bead:cell ratio of 1∶4 in the presence of 200 U/ml recombinant IL-2 (Proleukin S). After 4 days beads were removed and cells were maintained in complete RPMI 1640 medium containing 50 U/ml IL-2, 5 ng/ml IL-7 and 5 ng/ml IL-15 (ImmunoTools, Friesoythe, Germany) until starting the experiments.

### Cell Lines

PC3-PSCA cells, which permanently express PSCA after transduction [e. g. 14], were cultivated in complete RPMI 1640 medium supplemented with 10% FCS, 100 µg/ml penicillin/streptomycin, 1% non-essential amino acids, 2 mM N-acetyl-L-alanyl-L-glutamine and 1 mM sodium pyruvate (all supplied by Biochrom). 3T3 cells and HEK293T cells were cultivated in complete DMEM medium also supplemented with the same components like the RPMI 1640 medium with the exception of sodium pyruvate. All cell lines were maintained at 37°C in a humidified atmosphere of 5% CO_2_ [e. g. 18].

### Construction and Analysis of Novel Single-chain bsAbs

For redirection of T cells via the co-receptor CD8 a novel bsAb was constructed in the scBsTaFv format. The construct is based on the recently described humanized scBsTaFv CD3hu-PSCA(MB1) which is on the one hand directed to CD3 and on the other hand to PSCA a TAA which is expressed on the surface of prostate cancer cells [15], [21]. In principle, the anti-CD3 scFv domain in this scBsTaFv was replaced by the anti-CD8 scFv domain. The anti-CD8 scFv was cloned from a novel mAb to CD8 (CD8-MB10) which was established from hyperimmunized mice using conventional hybridoma technique [e. g. 22]. RNA was prepared from hybridoma cells which were four times subcloned from single cells as previously described [e. g. 14]. The cloning of mRNA sequences encoding the V_H_ and V_L_ regions of the mAbs was accomplished using highly degenerated primers for IgG1 isotypes as described previously [e. g. 22]. For amplification of the V_H_ chains, the following primer pair was used: IgG1-V_H_(for) 5′ GGATCCSARGTNMAGCTGSAGSAGTC 3′ and IgG1-V_H_(rev) 5′ GAARRCGTCGACCCTCCGCACCAGACCCTCCGCCACAGACCCTCCGCCACCATAGACAGATGGGGGTGTCTTTTGGC 3′. For amplification of the V_L_ chains, the following primer pair was used: IgG1-V_L_(for) 5′ GAATTCTGAYATTGTGMTSACMCAR WCTMCA 3′ and IgG1-V_L_(rev) 5′ GGGCCCGGATACAGTTGGTGCAGCATC-3′.

The murine (mu) anti-CD8 scFv was constructed in V_H_-V_L_ orientation using techniques previously described [e.g. 18]. For amplification and modification of anti-CD8 V_H_ the primer pair P1(for) 5′ GCCCAGCCGGCCGAAGTTAAGCTGCAGCAGTC 3′ and P2(rev) 5′ GGAGCCGCCGCCGCCAGAACC ACCACCACCAGAGACAGTGACCAGAGTCCC 3′ and for anti-CD8 V_L_ the primer pair P3(for) 5′ GGCGGCGGCGGCTCCGGTGGTGGTGGATCCGACATTGTGCT GACCCAGTCTCCA 3′ and P4(rev) 5′ GCGGCCGCGGATACAGTTGGTGCAGCATC 3′ was used. The PCR products of V_H_ and V_L_ were fused by overlapping PCR for which purpose the following primer pair was used: P5(for) 5′-TTACTCGCGGCCCAGCCGGCCATGGCGGA CTACAAAG 3′ and P6(rev) 5′ GGAGCCGCCGCCGCCAGAACCACCACCACCATAGACAGATGGGGGTGTCGT 3′. The PCR products were ligated into an *Sfi*I/*Not*I-restricted pSecTag2B vector. After plasmid amplification in *E. coli* Top10 and DNA purification the anti-CD8 scFv was excised by restriction enzymes *Sfi*I/*Not*I and ligated into the same restriction enzyme sites of the vector encoding the bispecific construct scBsTaFv CD3-PSCA(MB1) [15]. Thereby, the anti-CD3 scFv domain present in the original construct scBsTaFv CD3-PSCA(MB1) was replaced by the murine anti-CD8 scFv domain resulting in the novel scBsTaFv CD8-PSCA(MB1). A final schematic view of the newly generated bsAb construct including the N-terminal Igκ leader SP and the C-terminal myc- and his-tag is shown in **Fig. 1B**.

For permanent production the sequence of the novel recombinant bsAb including its N-terminal Igκ leader SP and C-terminal tags was cut from pSecTag2B by restriction enzymes *Nhe*I and *Mss*I and cloned into the lentiviral vector p6NST50, which was digested with *Xba*I/*Ksp*AI. All restriction enzymes were purchased from Fermentas (St. Leon-ROT, Germany). Finally, 3T3 cells were transduced with p6NST50 vector harboring the scBsTaFv sequence using a lentiviral packaging system as described previously [14], [23].

Alternatively, the bsAbs were produced by HEK293T cells after transient transfection with pSecTag2B harboring the scBsTaFv CD8-PSCA(MB1) sequence.

The recombinant his-tagged bsAbs were purified from cell culture supernatant and analyzed as described previously [14]. In brief, protein analysis was performed by SDS-PAGE and immunoblotting [e. g. 24, 25]. Therefore, isolated bsAbs were transferred onto a nitrocellulose membrane and detected by a murine anti-penta-his mAb (Quiagen, Hilden, Germany) and a rabbit anti-mouse-IgG conjugated with alkaline phosphatase (Dianova, Hamburg, Germany) using the substrate BCIP/NBT (Roche Diagnostics, Mannheim, Germany). Representative results of immunoblotting analysis and purity control with the help of a Coomassie Brilliant Blue G250 stained SDS-PAGE are presented in **Fig. 1C**.

Binding properties of recombinant Abs were analyzed by flow cytometry as described previously [14]. Briefly, antigen-positive cells were incubated with recombinant Abs and detected with anti-myc/FITC mAb (Miltenyi Biotec) using a FACSCalibur flow cytometer (BD Biosciences, Heidelberg, Germany).

### Immunofluorescence Microscopy

For epifluorescence microscopy 5×10^3^ PC3-PSCA tumor cells were cultivated with 5×10^4^ CD8+ T cells in the presence or absence of 30 pmol/ml scBsTaFv CD8-PSCA(MB1) in Nunc Lab-Tek Chamber Slides (ThermoFisher Scientific, Schwerte, Germany) for 22 h. Thereafter, medium was discarded and cells were fixed with 90% methanol for 10 minutes at −20°C and blocked with 5% human serum in 1× PBS for 15 min at 4°C. Finally, the scBsTaFv CD8-PSCA(MB1) was detected with anti-myc/FITC mAb (Miltenyi Biotec). Samples were washed and covered with DAPI-containing cover medium AKLIDES (Medipan, Dahewitz, Germany) and cover slides. Microscopic images were taken with a Zeiss Axiovert 200M epifluorescence microscope and edited with Axiovision software package (Zeiss, Jena, Germany).

### T cell Activation Assay and Cytokine ELISA

After 22 h-incubation of 5×10^3^ PC3-PSCA tumor cells with 5×10^4^ freshly isolated PBMCs or isolated CD8+ T cells in the presence or absence of 30 pmol/ml of recombinant Abs, cells were spun down and supernatants were collected. T cells of one triplet were pooled, stained with anti-CD25/PE, anti-CD69/PE-Cy5, and anti-CD8/FITC (Miltenyi Biotec GmbH) and measured using a flow cytometer. Cytokine concentrations in collected supernatants were determined using OptEIA Human IFN-γ and OptEIA Human TNF ELISA Kits (BD Biosciences).

### Cytotoxicity Assay

Standard chromium release assays were performed as described elsewhere [14], [18], [19]. Briefly, effector T cells were co-cultured with 5×10^3^ [^51^Cr]-labeled tumor cells at different effector to target cell (e:t) ratios for indicated time periods. Released [^51^Cr] was determined with the help of a beta counter (PerkinElmer Life Sciences, Rodgau-Rügesheim, Germany). If not stated otherwise, experiments were performed in triplicates for at least three donors and used for calculation of mean and standard deviation.

### Statistical Analysis

Statistical analysis was performed with GraphPad Prism software version 5.0 (GraphPad Software Inc., La Jolla, CA, USA) using non-parametric one-way ANOVA and Bonferroni multiple-comparison test. The p values *p<0.05 and ***p<0.001 were considered significant.

## References

[1] Kharfan-Dabaja MA, Hamadani M, Reljic T, Pyngolil R, Komrokji RS, et al (2013) Gemtuzumab ozogamicin for treatment of newly diagnosed acute myeloid leukaemia: a systematic review and meta-analysis. Br J Haematol 163: 315–325 10.1111/bjh.12528 24033280

[2] Pickhard A, Piontek G, Seidl C, Kopping S, Blechert B, et al (2014) ^1^ ^3^Bi-anti-EGFR radioimmunoconjugates and X-ray irradiation trigger different cell death pathways in squamous cell carcinoma cells. Nucl Med Biol 241: 68–76 10.1016/j.nucmedbio.2013.09.010 24210808

[3] Chelius D, Ruf P, Gruber P, Plöscher M, Liedtke R, et al (2010) Structural and functional characterization of the trifunctional antibody catumaxomab. MAbs 2: 309–319. 20418662 10.4161/mabs.2.3.11791 PMC2881257

[4] Müller D, Kontermann RE (2010) Bispecific antibodies for cancer immunotherapy: Current perspectives. BioDrugs 24: 89–98 10.2165/11530960-000000000-00000 20199124

[5] Stamova S, Koristka S, Keil J, Arndt C, Feldmann A, et al (2012) Cancer immunotherapy by retargeting of immune effector cells via recombinant bispecific antibody constructs. Antibodies 1: 172–198.

[6] Byrne H, Conroy PJ, Whisstock JC, O’Kennedy RJ (2013) A tale of two specificities: bispecific antibodies for therapeutic and diagnostic applications. Trends Biotechnol 31: 621–632. 24094861 10.1016/j.tibtech.2013.08.007 PMC7114091

[7] Hoffmann P, Hofmeister R, Brischwein K, Brandl C, Crommer S, et al (2005) Serial killing of tumor cells by cytotoxic T cells redirected with a CD19−/CD3-bispecific single-chain antibody construct. Int J Cancer 115: 98–104. 15688411 10.1002/ijc.20908

[8] Offner S, Hofmeister R, Romaniuk A, Kufer P, Baeuerle PA (2006) Induction of regular cytolytic T cell synapses by bispecific single-chain antibody constructs on MHC class I-negative tumor cells. Mol Immunol 43: 763–771. 16360021 10.1016/j.molimm.2005.03.007

[9] Baeuerle PA, Reinhardt C (2009) Bispecific T-cell engaging antibodies for cancer therapy. Cancer Res 69: 4941–4944. 19509221 10.1158/0008-5472.CAN-09-0547

[10] Stamova S, Feldmann A, Cartellieri M, Arndt C, Koristka S, et al (2012) Generation of single-chain bispecific green fluorescent protein fusion antibodies for imaging of antibody-induced T cell synapses. Anal Biochem 423: 261–268. 22274538 10.1016/j.ab.2011.12.042

[11] Nagorsen D, Kufer P, Baeuerle PA, Bargou R (2012) Blinatumomab: a historical perspective. Pharmacol Ther 136: 334–342. 22940266 10.1016/j.pharmthera.2012.07.013

[12] Koristka S, Cartellieri M, Arndt C, Bippes CC, Feldmann A, et al (2013) Retargeting of regulatory T cells to surface-inducible autoantigen La/SS-B. J Autoimmun 42: 05–16 10.1016/j.jaut.2013.01.002. Epub 2013 Jan 22 23352111

[13] Arndt C, Feldmann A, von Bonin M, Cartellieri M, Ewen EM, et al (2013) Costimulation improves the killing capability of T cells redirected to tumor cells expressing low levels of CD33: description of a novel modular targeting system. Leukemia Aug 20 10.1038/leu.2013.243. Epub 23958923

[14] Feldmann A, Stamova S, Bippes CC, Bartsch H, Wehner R, et al (2011) Retargeting of T cells to prostate stem cell antigen expressing tumor cells: comparison of different antibody formats. Prostate 71: 998–1011. 21541976 10.1002/pros.21315

[15] Feldmann A, Arndt C, Töpfer K, Stamova S, Krone F, et al (2012) Novel humanized and highly efficient bispecific antibodies mediate killing of prostate stem cell antigen-expressing tumor cells by CD8+ and CD4+ T cells. J Immunol 189: 3249–3259. 22875801 10.4049/jimmunol.1200341

[16] Dreier T, Baeuerle PA, Fichtner I, Grün M, Schlereth B, et al (2003) T cell costimulus-independent and very efficacious inhibition of tumor growth in mice bearing subcutaneous or leukemic human B cell lymphoma xenografts by a CD19−/CD3- bispecific single-chain antibody construct. J Immunol 170: 4397–4402. 12682277 10.4049/jimmunol.170.8.4397

[17] Pörtner LM, Schönberg K, Hejazi M, Brünnert D, Neumann F, et al (2012) T and NK cells of B cell NHL patients exert cytotoxicity against lymphoma cells following binding of bispecific tetravalent antibody CD19×CD3 or CD19×CD16. Cancer Immunol Immunother Oct;61(10): 1869–1875. Epub 2012 Sep 14 10.1007/s00262-012-1339-9 PMC11028742 22976535

[18] Arndt C, von Bonin M, Cartellieri M, Feldmann A, Koristka S, etal (2013) Redirection of T cells with a first fully humanized bispecific CD33-CD3 antibody efficiently eliminates AML blasts without harming hematopoietic stem cells. Leukemia 27[4]: 964–967. 10.1038/leu.2013.18 23325142

[19] Stamova S, Cartellieri M, Feldmann A, Bippes CC, Bartsch H, et al (2011) Simultaneous engagement of the activatory receptors NKG2D and CD3 for retargeting of effector cells to CD33-positive malignant cells. Leukemia: 25[6]: 1053–1056. 10.1038/leu.2011.42 21415850

[20] Grossman WJ, Verbsky JW, Tollefsen BL, Kemper C, Atkinson JP, Ley TJ (2004) Differential expression of granzymes A and B in human cytotoxic lymphocyte subsets and T regulatory cells. Blood Nov 1;104(9): 840–848. Epub 2004 Jul 6 10.1182/blood-2004-03-0859 15238416

[21] Cunha AC, Weigle B, Kiessling A, Bachmann M, Rieber EP (2006) Tissue-specificity of prostate specific antigens: comparative analysis of transcript levels in prostate and non-prostatic tissues. Cancer Lett 236: 229–238. 16046056 10.1016/j.canlet.2005.05.021

[22] Bippes CC, Feldmann A, Stamova S, Cartellieri M, Schwarzer A, et al (2011) A novel modular antigen delivery system for immuno targeting of human 6-sulfo LacNAc-positive blood dendritic cells (SlanDCs). PLoS One Jan 21 6(1): e16315 10.1371/journal.pone.0016315 PMC3025022 21283706

[23] Morgenroth A, Cartellieri M, Schmitz M, Gunes S, Weigle B, et al (2007) Targeting of tumor cells expressing the prostate stem cell antigen (PSCA) using genetically engineered T-cells. Prostate 67[10]: 1121–1131. 10.1002/pros.20608 17492652

[24] Bartsch H, Bachmann M (2009) Sequential use of immunoblots for characterization of autoantibody specificities. Methods Mol Biol 536: 293–298 10.1007/978-1-59745-542-8-31 19378068

[25] Arndt C, Koristka S, Feldmann A, Bartsch H, Bachmann M (2012) Coomassie-Brilliant Blue staining of polyacrylamide gels. Methods Mol Biol 869: 465–469 10.1007/978-1-61779-821-4-40. PMID: 22585511[PubMed - indexed for MEDLINE] 22585511

